# Metal artifact reduction sequence MRI in hip periprosthetic joint infection management: a noninvasive reference for infection control before two-stage reimplantation

**DOI:** 10.1302/2046-3758.159.BJR-2025-0586.R1

**Published:** 2026-09-04

**Authors:** Zhenyuan Lin, Lan Lin, Mingzhong Liu, Chaofan Zhang, Wenming Zhang, Yang Chen, Chaoyang Wu, Zida Huang, Zhenpeng Guan, Xinyu Fang

**Affiliations:** 1 Department of Orthopedics, The First Affiliated Hospital, Fujian Medical University, Fuzhou, China; 2 Department of Orthopaedic Surgery, National Regional Medical Center, Binhai Campus of the First Affiliated Hospital, Fujian Medical University, Fuzhou, China; 3 Department of Orthopedic Surgery, Quanzhou First Affiliated Hospital of Fujian Medical University, Quanzhou, China; 4 Orthopedics Department, Peking University Shougang Hospital, Beijing, China

**Keywords:** Periprosthetic joint infection, MRI, MARS, Infection control diagnosis, Two-stage reimplantation, metal, MRI scanning, oedema, hip, infections, periprosthetic joint infections (PJIs), debridement, CRP, soft tissues, Musculoskeletal Infection

## Abstract

**Aims:**

There is currently no validated noninvasive method to determine infection control prior to prosthesis reimplantation during the two-stage revision process for hip periprosthetic joint infections (PJI) after total hip arthroplasty (THA). This prospective study investigates whether metal artifact reduction sequence (MARS) MRI can serve as a diagnostic reference for infection control before second-stage revision surgery by analyzing artifact-reduced MRIs in patients with hip PJI prior to reimplantation.

**Methods:**

Patients with hip PJI after THA who entered the two-stage revision process between January 2019 and January 2024 were prospectively and consecutively enrolled. Based on the Musculoskeletal Infection Society (MSIS) criteria, 23 patients achieved infection control after the interval period (prior to reimplantation), and 11 experienced infection recurrence after spacer implantation (prior to repeat debridement). All patients underwent MARS MRI prior to both first-stage surgery and second-stage reimplantation. Data collected included patient demographics (sex, age), ESR, CRP, intraoperative pathology, culture results, and MARS MRIs. Two imaging specialists reviewed the MARS MRIs obtained prior to the first-stage surgery and prior to the second-stage reimplantation, evaluating regression of prior infectious lesions, emergence of new lesions, and overall infection control. Their assessments were then compared with intraoperative pathology and culture-confirmed infection status.

**Results:**

MARS MRI revealed significantly more frequent findings of bone oedema, soft-tissue oedema, and joint effusion in patients with recurrent infection compared to those with controlled infection after spacer implantation (p = 0.002). The imaging specialists’ evaluations were in complete agreement with the clinical findings confirmed by pathology and cultures.

**Conclusion:**

Evaluating the difference in MARS MRIs between the first-stage and second-stage preoperative periods, and focusing on oedema signals in the bone and soft tissues surrounding the prosthesis, provides a useful noninvasive indicator for determining infection control prior to two-stage reimplantation.

Cite this article: *Bone Joint Res* 2026;15(9):1101–1110.

## Article focus

- This prospective study investigates whether metal artifact reduction sequence (MARS) MRI can act as a reliable, noninvasive diagnostic reference for assessing infection control before second-stage reimplantation in patients with hip periprosthetic joint infections (PJIs).
- The research evaluates how the diagnostic accuracy of MARS MRI compares to traditional Musculoskeletal Infection Society (MSIS) diagnostic indicators prior to a two-stage revision.
- The study aims to identify which specific MARS MRI signs (specifically bone oedema, periprosthetic tissue oedema, and joint cavity effusion) are indicative of an active infection prior to reimplantation.

## Key messages

- MARS MRI demonstrates high reliability as a noninvasive tool for evaluating infection control, offering a diagnostic sensitivity (78.26%) that is comparable to conventional methods such as CRP, ESR, and intraoperative cultures.
- Patients experiencing infection recurrence exhibit significantly more findings of bone oedema, soft-tissue oedema, and joint effusion on MARS MRI compared to patients who have successfully achieved infection control.
- Analyzing the dynamic changes in MARS MRIs between the first-stage and second-stage preoperative periods provides orthopaedic surgeons with valuable reference data to guide the optimal timing for reimplantation.

## Strengths and limitations

- The study prospectively addresses a core clinical dilemma by proposing a novel, noninvasive alternative to invasive traditional tests for determining infection control specifically at the reimplantation stage.
- The relatively small sample size (34 patients) from a single centre compromises statistical power, restricts the ability to perform subgroup analyses, and may introduce selection bias that limits the generalizability of the findings.

## Introduction

Total hip arthroplasty (THA) is the standard treatment for end-stage hip disease, but periprosthetic joint infection (PJI) remains a serious complication.^1^ With global THA demand projected to rise steadily from the 2000s to 2030, PJI cases are expected to increase accordingly.^2^

For chronic PJI > four weeks after surgery or > four weeks from the onset of acute infection to effective diagnosis and treatment, two-stage revision is the widely accepted treatment.^3^ This involves a first-stage surgery, i.e., debridement, removal of the prosthesis, implantation of a cemented spacer containing antibiotics (some of them contain metal), and antibiotic application (intravenous and oral) for about six to 12 weeks,^4^ and judgement of infection control prior to proceeding to a second-stage procedure, i.e., reimplantation of a new prosthesis, or else repeat debridement should be considered. Unfortunately, the failure rate of second-stage revision is still 12% to 25%.^5,6^

Failure of two-stage revision is associated with various factors, including patient demographics, disease characteristics, and treatment protocols.^7,8^ A key dilemma remains the inability to accurately assess infection control prior to two-stage reimplantation. Currently, no validated noninvasive method with high sensitivity and specificity exists for this purpose.^9,10^ The Musculoskeletal Infection Society (MSIS) criteria,^11,12^ although widely accepted for diagnosing PJI, are designed for initial diagnosis rather than assessing infection resolution prior to reimplantation, limiting their sensitivity in this context.^13,14^ Pre-reimplantation arthrocentesis, as referenced by the ICM, provides certain diagnostic value; however, it is invasive and entails a potential risk of introducing new infection.^15^ Intraoperative tissue culture, a necessary test, has a too-low culture positivity rate due to the application of antibiotic-containing bone cement.^16^ Moreover, synovial or tissue cultures cannot be performed immediately intraoperatively, and even if the culture is positive, the reimplantation procedure is already over,^17^ thus failing to provide convincing support at the time of reimplantation; orthopaedic surgeons need to obtain more reference data preoperatively.

MRI is highly accurate for septic infections and periprosthetic osteolysis,^18^ and is a noninvasive diagnostic method. However, artifacts produced by metal-containing spacers limit the use of conventional serial MRI to reflect the true level of inflammation in soft tissues accurately. With the recent development of MRI sequence technology, many strategies have been effective in reducing metal artifacts in MRI, such as high bandwidth sequences, view angle tilling, multi-acquisition variable resonance image combination, slice encoding for metal art correction, etc. They are referred to as metal artifact reduction sequences (MARS).^19^ The ability of MARS to reduce artifacts and to show the metal-bone interface, bone, and soft tissues around the prosthesis makes its application in orthopaedic implant-related infections possible.^20-22^ To the best of our knowledge, there are no studies that have used MARS MRI as a diagnostic reference for infection control prior to the two-stage revision of hip PJI.

Therefore, the aims of our study were: 1) how does the diagnostic accuracy of MARS MRI compare to MSIS-related diagnostic indicators in hip PJI prior to reimplantation of a two-stage revision?; and 2) which signs of MARS MRI are indicative of the presence of infection in hip PJI prior to two-stage reimplantation?

## Methods

### Participant selection and clinical data collection

**Fig. 1:**
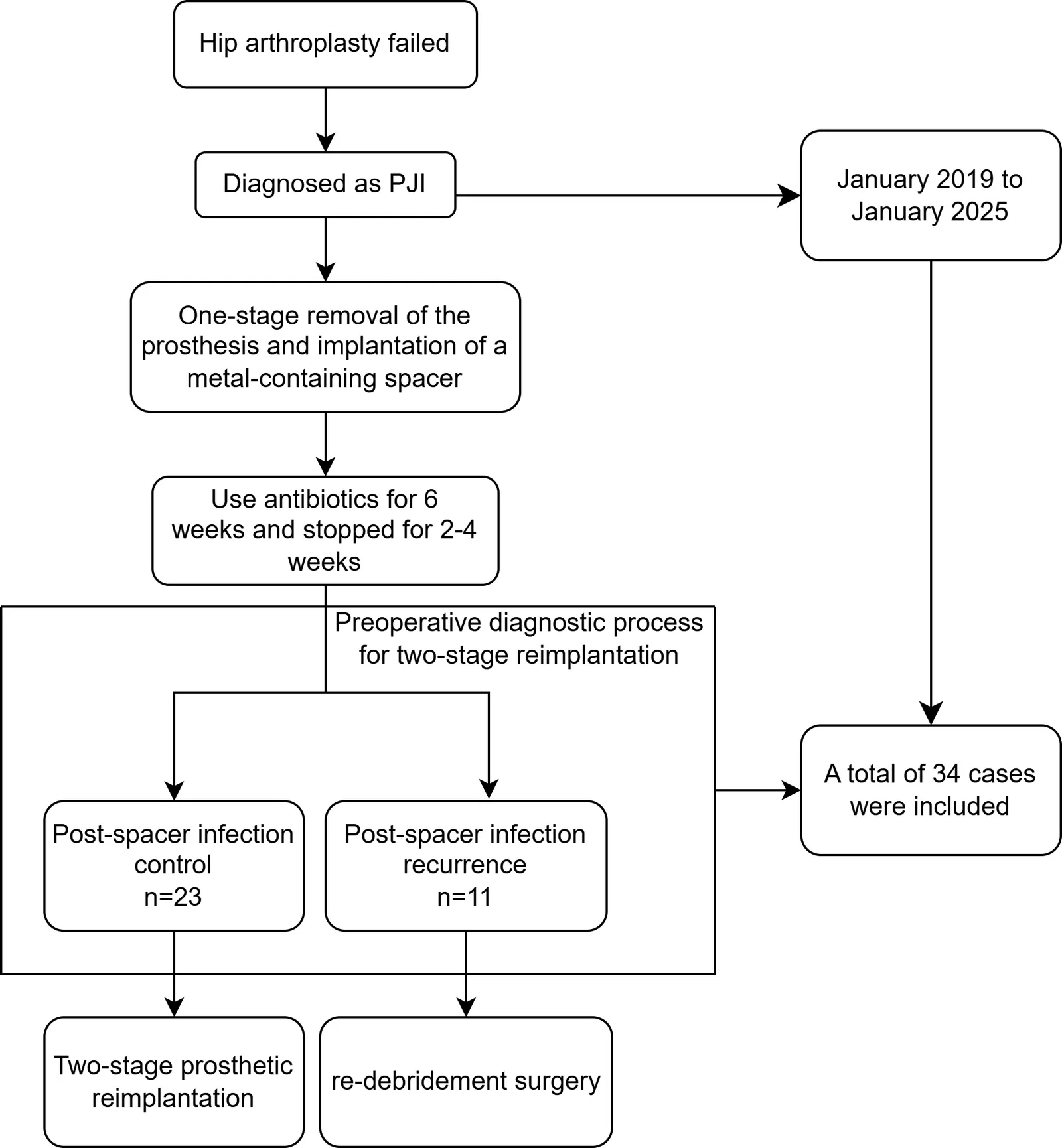
Flowchart of hip two-stage revision and inclusion of cases. PJI, periprosthetic joint infection.

Patients with hip PJI who underwent two-stage revision surgery between January 2019 and January 2024 at our hospital were prospectively collected. The inclusion criteria were patients with hip PJI who had undergone a two-stage revision and first-stage and second-stage surgery; implantation of metal-containing spacers at the time of first-stage surgery; and those who underwent MARS MRI scanning prior to one-stage surgery and two-stage reimplantation. The case exclusion criteria were patients with incomplete case data; incomplete MARS MRI or substandard image quality; or who were lost to follow-up. Overall, 34 cases were included, and five cases were excluded. Among them, four cases had incomplete MARS MRI data, and one case was lost to follow-up. Data on ESR, CRP, synovial fluid white blood cell count (SF-WBC), and synovial fluid polymorphonuclear neutrophil percentage (SF-PMN%), joint fluid, tissue culture, pathology slides, and antibiotic application regimen were also collected from patients prior to the two-stage reimplantation. The diagnosis of hip PJI was based on the MSIS criteria,^11,12^ and the definition of treatment failure was based on the Delphi-based international multidisciplinary consensus.^23^ The case inclusion flowchart is shown in Figure 1.

A total of 34 patients were included in this study, all of whom underwent MARS MRI scans prior to both the first-stage and second-stage revision surgeries. The general data of the patients are shown in Table I. Data on radiograph, ESR, CRP, SF-WBC, SF-PMN%, joint fluid, tissue culture, pathology slides, and antibiotic application regimen were collected from the patients.

**Table I.** Characteristics of cases.

Characteristic	All cases	Post-spacer infection control	Post-spacer infection recurrence	p-value
Male, n	22	15	7	> 0.999*
Mean age, yrs (SD)	58.470 (11.373)	56.520 (11.020)	62.550 (11.518)	0.151†
**Joint involved, n**				> 0.999*
Left hip	15	10	5	
Right hip	19	13	6	
Median CRP, mg/L (IQR)	5.000 (3.470 to 24.253)	3.870 (2.650 to 5.000)	47.900 (16.000 to 102.000)	< 0.001‡
Mean ESR, mm/h (SD)	42.029 (37.581)	23.913 (20.268)	79.909 (37.732)	< 0.001†
Median SF-WBC, × 10^6^ /L (IQR)	1,447.000 (860.000 to 7,102.750)	980.000 (634.000 to 1,459.000)	12,862.000 (5,857.000 to 138,811.000)	< 0.001‡
Median SF-PMN%, % (IQR)	55.550 (33.950 to 80.400)	46.100 (30.600 to 58.300)	84.400 (80.300 to 93.800)	< 0.001‡

* Chi-squared test.

† Independent-samples *t*-test.

‡ Mann-Whitney U test.

PJI, periprosthetic joint infection; SF-PMN, synovial fluid polymorphonuclear; SF-WBC, synovial fluid white blood cell.

### Demographics of cases

The 34 patients in this study included 22 males and 12 females, with a mean age of 58.47 years (31 to 79). Among them, 15 had left hip PJI and 19 had right hip PJI. According to the MSIS criteria, there were no statistically significant differences between the 23 patients with post-spacer infection control and the 11 patients with post-spacer infection recurrence in terms of sex, age, and site of PJI (p > 0.05). However, there were significant differences in CRP, ESR, SF-WBC, and SF-PMN% between the two groups (p < 0.001). The relevant data are presented in Table I.

The study was approved by the Ethics Committee of the First Affiliated Hospital of Fujian Medical University, and all patients gave informed consent and signed an informed consent form.

### Surgical procedure of two-stage revision

For patients diagnosed with PJI following hip arthroplasty, the first stage involves removal of the prosthesis, thorough debridement of necrotic and infected tissue, and repeated irrigation with hydrogen peroxide, povidone-iodine, and saline. An antibiotic-loaded bone cement spacer is then prepared according to the results of microbial culture and drug susceptibility testing; in culture-negative cases, vancomycin (2.0 g) and meropenem (1.0 g) are empirically added per 40 g of bone cement. In the hip, prosthesis-like spacers are most commonly used. Postoperatively, patients receive systemic intravenous antibiotics for approximately six weeks, followed by a two- to four-week antibiotic-free interval before reimplantation assessment.

The evaluation prior to the second stage includes MARS MRI, synovial fluid white blood cell count, polymorphonuclear neutrophil percentage, microbial culture, and susceptibility testing. During the second-stage procedure, at least five periprosthetic tissue specimens are obtained for culture and histopathological examination. Once infection control is confirmed, reimplantation is performed: the spacer is removed, radical debridement and irrigation are repeated, and a new prosthesis is implanted to complete reconstruction.

### MARS protocol

A 1.5 T MRI device (Skyra, Siemens Healthcare, Germany) was applied for scanning. The patient was placed supine on the scanning bed and scanned so that the imaging plane was parallel to the long axis of the prosthesis. No intravenous contrast enhancement was administered in any MRI examinations in this study, as the diagnostic assessment focused on the intrinsic signal changes of oedema and effusion. Scanning for metal removal artifacts was performed using MARS sequence protocols. MARS-MRI was conducted using the optimized parameters at 1.5 T in a coronal and axial STIR (short-tau-inversion recovery), a non-fat-saturated T2 in coronal view in the 34 patients. Imaging protocols were standardized using the following sequences: Coronal short tau inversion recovery (STIR) with repetition time (TR) 4220 ms, echo time (TE) 36 ms, 4 mm slice thickness, and 340 × 340 mm^2^ field of view; transverse STIR employing TR 4,000 ms, TE 31 ms, 4 mm slice thickness, and 360 × 270 mm^2^ field of view. Additional acquisitions included coronal non-fat-saturated T2-weighted imaging (TR 4,000 ms, TE 48 ms, 4 mm slice thickness, 360 × 360 mm^2^ field of view). Minor adjustments were made to some parameters as needed.

### Definition of MARS characteristic performance on hip PJI

**Fig. 2:**
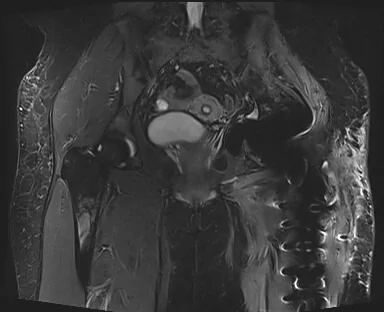
Conventional MRI sequences show obvious artifacts, making it impossible to evaluate the periprosthetic tissues.

**Fig. 3:**
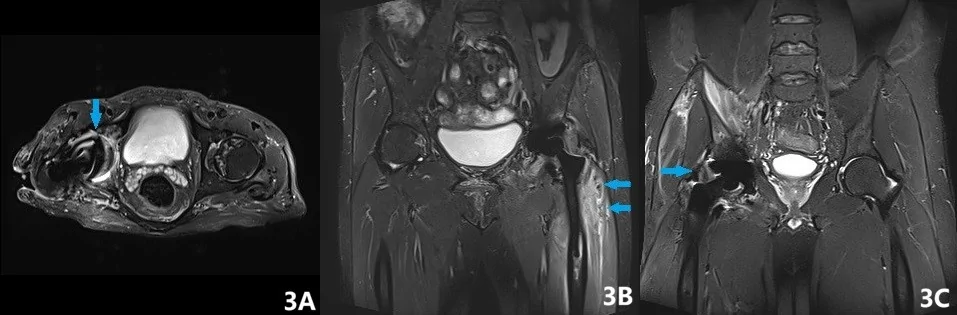
a) Bone oedema; b) periprosthetic tissue oedema; and c) effusion in the joint cavity.

As reported in the literature,^24-26^ metal artifacts of conventional MRI sequences restrict the accurate evaluation of soft-tissue inflammation (Figure 2). After excluding metal artifacts, hip PJI was characterized on MARS MRI as follows: bone oedema (Figure 3a): high signal of bone on the suppressed fat sequence T2; periprosthetic tissue oedema (Figure 3b, juxtaposed arrow): high signal of periprosthetic tissue on the suppressed fat T2 sequence; and effusion in the joint cavity (Figure 3c): high signal of effusion in the joint cavity.

### MARS MRI analysis

An orthopaedic PJI specialist (WZ) with more than 30 years of experience, and a musculoskeletal radiologist (CW) with more than 20 years of practice, first evaluated the presence of bone oedema, periprosthetic tissue oedema, and intra-articular joint cavity effusion on MARS MRI, and then compared these images prior to the first-stage surgery and reimplantation of second-stage to determine if the infection of the diseased joint was controlled. When there was a disagreement, a unanimous opinion was obtained by consultation. The patient information they obtained on the MARS MRI was masked. At two weeks after the end of the first judgement, the order of all MARS MRIs was shuffled, and the persistence of the infection was judged again to evaluate the assessor’s assessment stability. The results of the first assessment were used to assess the diagnostic accuracy of the MARS MRI.

### Statistical analysis

Normally distributed variables are described by mean (SD), and non-normally distributed variables are described by median (IQR). Categorical variables were expressed as counts and proportions.

Statistical differences were analyzed using independent-samples *t*-tests, chi-squared tests, Fisher’s exact test, or Mann-Whitney U tests, depending on the characteristics of the variables. The intraobserver reliability was analyzed by κ-test. The McNemar test was used to assess differences in diagnostic outcomes between patients’ MARS MRI images and conventional tests (culture, CRP, ESR, SF-WBC, and SF-PMN%).

All reported p-values were the result of a two-sided test and were considered statistically significant at p < 0.05. Statistical analysis was performed using SPSS v. 26.0 (IBM, USA).

## Results

There were 20 pathogen culture-negative and three culture-positive cases in patients with post-septal infection control (three intraoperative cultures were positive for a single sample, which was considered to be a contaminated specimen, and no recurrence of the infection has been observed so far at nine and 12 months of postoperative follow-up). There were two culture-negative and nine culture-positive patients with recurrence of infection after the spacer. The microbiological data of the patients prior to two-stage reimplantation surgery, or prior to reinfection debridement surgery, are shown in Table II.

**Table II.** Microbiology culture findings.

Post-spacer infection control, n = 23	Post-spacer infection recurrence, n = 11
**Culture-positive, n = 3**	**Culture-positive, n = 9**
*Staphylococcus aureus*, n = 1	*Staphylococcus aureus*, n = 4
*Klebsiella pneumoniae*, n = 1	*Pseudomonas aeruginosa*, n = 2
*Staphylococcus hominis*, n = 1	Near-smooth Candida complex, n = 1
	*Staphylococcus epidermidis*, n = 1 *Staphylococcus pasteuri*, n = 1
**Culture-negative, n = 20**	**Culture-negative, n = 2**

### MARS MRI features of hip PJI and its diagnostic value

Among the MARS sequence MRI features, hip PJI patients with post-spacer infection recurrence (prior to reinfection debridement surgery) had a higher probability of developing bone oedema (p < 0.001), periprosthetic tissue oedema (p < 0.001), and effusion in the joint cavity (p < 0.001, all Fisher’s exact test) than hip PJI patients with post-spacer infection control (prior to the two-stage reimplantation surgery). Bone oedema had the highest specificity (90.91%) with a positive predictive value of 95.00% and a negative predictive value of 71.43%. Joint cavity effusion had the highest sensitivity (86.96%) with a positive predictive value of 86.96% and a negative predictive value of 72.73%. The relevant data are shown in Table III.

**Table III.** Statistical analysis of the diagnostic efficiency of each index of Musculoskeletal Infection Society criteria and metal artifact reduction sequence (MARS) MRI in periprosthetic joint infection.

Variable	Post-spacer infection control	Post-spacer infection recurrence	p-value*	Sensitivity, %	p-value (vs MARS)	Specificity, %	p-value (vs MARS)	Youden’s Index	PPV, %	NPV, %	LR+	LR-
MARS MRI	18/23	2/11	0.002	78.26	N/A	81.82	N/A	0.601	90.00	64.29	4.304	0.266
Culture	20/23	2/11	< 0.001	86.96	0.625	81.82	> 0.999	0.688	90.91	75.00	4.783	0.159
Preop culture	22/23	6/11	0.008	95.65	0.219	45.45	0.219	0.411	78.57	83.33	1.754	0.096
Intraop culture	20/23	2/11	< 0.001	86.96	0.625	81.82	> 0.999	0.688	90.91	75.00	4.783	0.159
CRP (< 10 mg/L)	22/23	1/11	< 0.001	95.65	0.219	90.91	> 0.999	0.866	95.65	90.91	10.522	0.048
ESR (< 30 mm/h)	15/23	2/11	0.026	65.22	0.453	81.82	> 0.999	0.470	88.24	52.94	3.587	0.425
SF-WBC (< 3,000 × 106 /L)	21/23	1/11	< 0.001	91.30	0.375	90.91	> 0.999	0.822	95.45	83.33	10.043	0.096
SF-PMN% (< 80%)	23/23	2/11	< 0.001	100.00	N/A	81.82	> 0.999	0.818	92.00	N/A	5.500	0.000
Bone oedema	19/23	1/11	< 0.001	82.61	N/A	90.91	N/A	0.735	95.00	71.43	9.087	0.191
Joint cavity effusion	20/23	3/11	0.001	86.96	N/A	72.73	N/A	0.597	86.96	72.73	3.188	0.179
Soft-tissue oedema	19/23	2/11	< 0.001	82.61	N/A	81.82	N/A	0.644	90.48	69.23	4.543	0.213

Youden’s index = sensitivity + specificity − 1; LR+: Positive likelihood ratio, LR−: Negative likelihood ratio.

* Fisher’s exact test.

N/A, not applicable; NPV, negative predictive value; PPV, positive predictive value.

### Diagnostic efficacy of MARS MRI

A κ-test was performed on the evaluators’ two diagnoses, and the κ-value was 0.71 (95% CI 0.58 to 0.85; p < 0.001), which made the evaluation robust. The sensitivity of MARS MRI to diagnose whether the infection was controlled after the spacer was 78.26%, and the difference was statistically non-significant when compared with culture, CRP, ESR, SF-WBC, and SF-PMN%; the specificity was 81.82%, and the difference was not statistically significant compared with culture, CRP, ESR, and SF-WBC; the Youden’s Index was 0.601, lower than culture (0.688), CRP (0.866), and SF-WBC (0.822), and SF-PMN% (0.818), higher than ESR (0.470). The relevant data are shown in Table III.

### Typical case

**Fig. 4:**
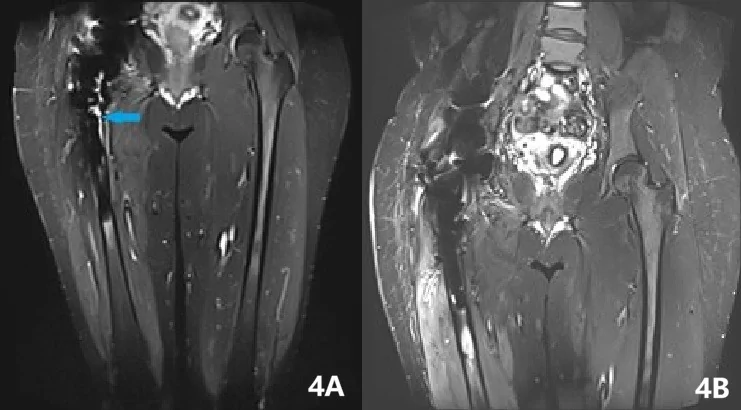
a) Preoperative metal artifact reduction sequence (MARS) sequence of the right hip debridement, with metal-bone interface infection manifestations indicated by the blue arrow. b) Review MRI MARS sequence three months after debridement.

The first case, shown in Figure 4, is a 36-year-old female with right hip pain for more than one year, which began more than one month after surgery accompanied by a week-long fever. The diagnosis was PJI of the right hip. Three months after debridement, there was soft-tissue inflammatory oedema around the prosthesis, femoral oedema was slightly reduced, soft-tissue inflammatory oedema high signal was still present, and the infection was not completely controlled.

**Fig. 5:**
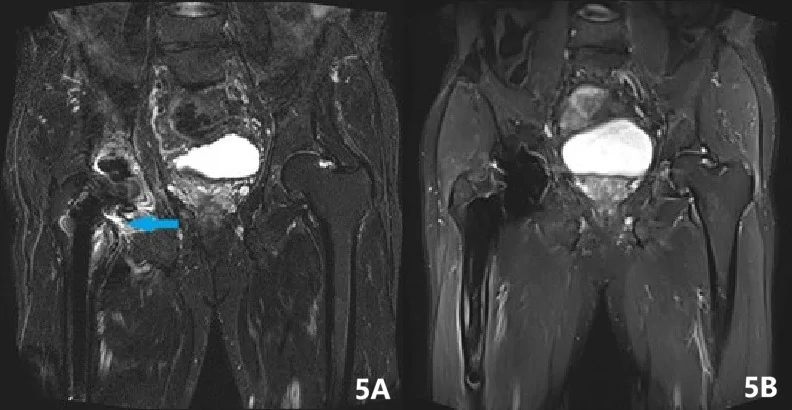
a) Preoperative metal artifact reduction sequence (MARS) MRI sequence image of the right hip debridement in a 70-year-old male, showing inflammatory oedema in the periprosthetic tissue as indicated by the blue arrow. b) Ten months after debridement surgery performed in the second‑stage procedure, pre‑reimplantation MARS‑MRI sequences were obtained.

The second case, shown in Figure 5, is a 70-year-old male, 15 years after right-sided hip arthroplasty, with recurrent oozing from the incision for nine years, which was then aggravated for three months. The diagnosis was PJI of the right hip. Ten months after the debridement surgery, pre‑reimplantation MARS MRI sequences showed no obvious inflammatory or infectious signals in the periprosthetic tissue, and intraoperative tissue cultures obtained during the second‑stage surgery were negative for pathogens. The intraoperative pathology showed that the neutrophils in the lesion area were < five high-power fields (HPFs), which was consistent with the preoperative images.

## Discussion

Our findings indicate that MARS MRI demonstrates high reliability in assessing infection control prior to reimplantation during two-stage revision procedures. Its overall diagnostic performance was comparable to conventional methods, including CRP, ESR, WBC count, PMN%, and intraoperative cultures, while it provided superior accuracy in detecting bone and soft-tissue oedema. These results suggest that MARS MRI may serve as a valuable noninvasive tool for guiding the optimal timing of reimplantation.

Despite extensive efforts, there is still no consensus on how to accurately determine infection eradication before reimplantation in clinical practice. Traditional approaches rely mainly on clinical manifestations and serological markers such as CRP and ESR. However, their diagnostic accuracy for persistent infection is limited, partly due to the confounding effects of prolonged antibiotic or anti-inflammatory therapy. In previous studies, CRP achieved a sensitivity of 85.7% and specificity of 81.0%, whereas ESR showed lower diagnostic value, with a specificity of only 64.3%.^27-30^

Alternative approaches such as synovial fluid biomarkers (e.g., calprotectin, α-defensin, interleukin-6) have shown high diagnostic accuracy in the initial diagnosis of PJI, however their use at the reimplantation stage is restricted. Only approximately 20% of patients provide adequate synovial fluid samples, and their diagnostic performance in this setting is unsatisfactory.^31-34^ Intraoperative frozen section analysis offers high specificity (94%) but limited sensitivity (50%), reducing its utility in excluding persistent infection.^27^ Similarly, intraoperative tissue cultures show low positive rates (approximately 15% to 20%), even with the aid of ultrasound sonication.^35-37^ Numerous studies have confirmed the diagnostic value of MRI in PJI. Galley et al^26^ validated the diagnostic accuracy of MARS MRI in detecting PJI-related periosteal reactions, cysts, and intramuscular oedema in their retrospective study on THA. Huang et al^38^ also identified occult lesions via preoperative MARS MRI in patients with PJI following THA and total knee arthroplasty. Existing data demonstrate that MRI yields a sensitivity of 76.3% to 100% and a specificity of 73.1% to 95% in detecting PJI-related oedema.^39-42^ However, most previous studies have focused on the initial diagnosis of PJI and not extended the application of MRI features to the assessment of infection control prior to two-stage reimplantation for hip PJI, leaving a lack of effective noninvasive assessment tools for this critical clinical juncture in clinical practice.^38,41^ Based on this, we conducted a prospective study that applied MARS MRI to the dynamic imaging assessment of patients with hip PJI. By comparing MARS MRIs obtained before the first-stage surgery and prior to two-stage reimplantation, we focused on the signal changes of periprosthetic bone oedema, soft-tissue oedema, and joint cavity effusion, and took the dynamic changes of these features as indicators for assessing infection control status, thus addressing the core clinical challenge of determining infection control status before two-stage reimplantation for hip PJI in a targeted manner. The results of this study confirmed that MARS MRI achieves satisfactory diagnostic sensitivity in detecting soft-tissue infections of hip PJI, which provides a novel noninvasive reference for the clinical assessment of infection control prior to two-stage reimplantation for hip PJI.

In this study, we focused on three MARS MRI features – bone oedema, soft-tissue oedema, and joint cavity effusion – which are readily identifiable even by non-specialist musculoskeletal radiologists. The combined evaluation of MARS sequence findings showed a statistically significant difference between patients with infection control after spacer implantation (prior to reimplantation) and those with infection recurrence (prior to repeat debridement) (p = 0.002). The effect of MARS MRI in the diagnosis of the sensitivity of the diagnosis of infection in hip PJI patients prior to two-stage reimplantation surgery was 78.26%, and there was no statistically significant difference when comparing with the conventional diagnostic indexes, such as CRP, ESR, SF-WBC, SF-PMN%, and intraoperative tissue culture (p > 0.05), which demonstrated that the diagnosis of infection by MARS MRI was no less effective than the commonly used diagnostic indexes. Further, MARS MRI is a noninvasive test that can be stably acquired prior to the two-stage revision surgery of hip PJI. In the cases shown in Figure 4 and Figure 5, the imaging findings were consistent with the corresponding pathological results, and findings on the MARS sequence might provide a reference for the ensuing clearance procedure. In one case of a patient with infection control after the interval period, although intraoperative tissue cultures were positive, we did not extend the duration of postoperative antibiotic therapy based on the MSIS criteria and MARS MRI findings. No infection recurrence was observed at nine- and 12-month follow-ups. This case highlights the value of MARS MRI in assisting surgeons in determining whether infection is controlled prior to two-stage reimplantation. While the assessment of infection using MARS MRI remains subjective and lacks absolute thresholds, this is similar to the interpretation of intraoperative frozen sections in pathology. When characteristic features are present, they can meaningfully support the diagnostic process.

This study has several limitations. First, although joint fluid biomarkers have demonstrated good diagnostic accuracy, they are not routinely tested at our institution; therefore, a comparison between MARS sequence MRI and these biomarkers was not possible. Second, the sample size was relatively small due to the low prevalence of hip PJI, which compromises the study’s statistical power. It limits subgroup analyses (e.g., by pathogenic bacteria, metal spacer type, or antibiotic therapy duration) and the detection of subtle but meaningful correlations between MARS MRI features and clinical outcomes. Moreover, this single-centre, small-sample study may have selection bias, making the findings less generalizable to other centres with different patient populations, surgical protocols, and MARS MRI scanning parameters. A multicentre study with a larger sample size may be necessary in the future to validate these findings.

MARS MRI is a useful noninvasive tool for evaluating infection control prior to reimplantation in two-stage revision for hip PJI. Changes in bone oedema, soft-tissue oedema, and joint effusion on sequential scans provide reliable indicators of infection status, with diagnostic value comparable to conventional markers.

## Data Availability

The authors confirm that the data supporting the findings of this study are available within the article.

## References

[1] Kapadia BH Berg RA Daley JA Fritz J Bhave A Mont MA Periprosthetic joint infection Lancet 2016 387 10016 386 394 10.1016/S0140-6736(14)61798-0 26135702

[2] Ahmed SS Haddad FS Prosthetic joint infection Bone Joint Res 2019 8 11 570 572 10.1302/2046-3758.812.BJR-2019-0340 31832177 PMC6888735

[3] Parvizi J Adeli B Zmistowski B Restrepo C Greenwald AS Management of periprosthetic joint infection: the current knowledge: AAOS exhibit selection J Bone Joint Surg Am 2012 94-A 14 e104 10.2106/JBJS.K.01417 22810411

[4] Matthews PC Berendt AR McNally MA Byren I Diagnosis and management of prosthetic joint infection BMJ 2009 338 b1773 10.1136/bmj.b1773 19482869

[5] Kheir MM Tan TL Gomez MM Chen AF Parvizi J Patients with failed prior two-stage exchange have poor outcomes after further surgical intervention J Arthroplasty 2017 32 4 1262 1265 10.1016/j.arth.2016.10.008 27838014

[6] Mortazavi SMJ Vegari D Ho A Zmistowski B Parvizi J Two-stage exchange arthroplasty for infected total knee arthroplasty: predictors of failure Clin Orthop Relat Res 2011 469 11 3049 3054 10.1007/s11999-011-2030-8 21866421 PMC3183212

[7] Kandel CE Jenkinson R Daneman N et al. Predictors of treatment failure for hip and knee prosthetic joint infections in the setting of 1- and 2-stage exchange arthroplasty: a multicenter retrospective cohort Open Forum Infect Dis 2019 6 11 ofz452 10.1093/ofid/ofz452 31737739 PMC6847009

[8] Bhanushali A Tran L Nairne-Nagy J et al. Patient-related predictors of treatment failure after two-stage total hip arthroplasty revision for periprosthetic joint infection: a systematic review and meta-analysis J Arthroplasty 2024 39 9 2395 2402 10.1016/j.arth.2024.04.053 38677343

[9] Tsai ML Herng-Shouh Hsu A Wu CT Lin PC Tan TL Kuo FC Optimal reimplantation timing in two-stage exchange for periprosthetic joint infection: an observative cohort study in Asian population BMC Musculoskelet Disord 2024 25 1 28 10.1186/s12891-023-07129-8 38166999 PMC10763399

[10] Khan IA Boyd BO Chen AF et al. Utility of diagnostic tests before reimplantation in patients undergoing 2-stage revision total joint arthroplasty: a systematic review and meta-analysis JBJS Rev 2023 11 3 36947634 10.2106/JBJS.RVW.22.00201 36947634

[11] Parvizi J Gehrke T Chen AF Proceedings of the International consensus on periprosthetic joint infection Bone Joint J 2013 95-B 11 1450 1452 10.1302/0301-620X.95B11.33135 24151261

[12] Parvizi J Tan TL Goswami K et al. The 2018 definition of periprosthetic hip and knee infection: an evidence-based and validated criteria J Arthroplasty 2018 33 5 1309 1314 10.1016/j.arth.2018.02.078 29551303

[13] Frangiamore SJ Siqueira MBP Saleh A Daly T Higuera CA Barsoum WK Synovial cytokines and the MSIS criteria are not useful for determining infection resolution after periprosthetic joint infection explantation Clin Orthop Relat Res 2016 474 7 1630 1639 10.1007/s11999-016-4710-x 26821163 PMC4887354

[14] Fillingham YA Della Valle CJ Suleiman LI et al. Definition of successful infection management and guidelines for reporting of outcomes after surgical treatment of periprosthetic joint infection: from the workgroup of the Musculoskeletal Infection Society (MSIS) J Bone Joint Surg Am 2019 101-A 14 e69 10.2106/JBJS.19.00062 31318814

[15] Aalirezaie A Bauer TW Fayaz H et al. Hip and knee section, diagnosis, reimplantation: proceedings of international consensus on orthopedic infections J Arthroplasty 2019 34 2S S369 S379 10.1016/j.arth.2018.09.021 30343965

[16] Chen AF Parvizi J Antibiotic-loaded bone cement and periprosthetic joint infection J Long Term Eff Med Implants 2014 24 2–3 89 97 10.1615/jlongtermeffmedimplants.2013010238 25272207

[17] Theil C Freudenberg SC Gosheger G Schmidt-Braekling T Schwarze J Moellenbeck B Do positive cultures at second stage re-implantation increase the risk for reinfection in two-stage exchange for periprosthetic joint infection? J Arthroplasty 2020 35 10 2996 3001 10.1016/j.arth.2020.05.029 32546394

[18] Gao Z Jin Y Chen X et al. Diagnostic value of MRI lamellated hyperintense synovitis in periprosthetic infection of hip Orthop Surg 2020 12 6 1941 1946 10.1111/os.12789 33225607 PMC7767676

[19] Olsen RV Munk PL Lee MJ et al. Metal artifact reduction sequence: early clinical applications Radiographics 2000 20 3 699 712 10.1148/radiographics.20.3.g00ma10699 10835123

[20] Chen CA Chen W Goodman SB et al. New MR imaging methods for metallic implants in the knee: artifact correction and clinical impact J Magn Reson Imaging 2011 33 5 1121 1127 10.1002/jmri.22534 21509870 PMC3081101

[21] Jungmann PM Agten CA Pfirrmann CW Sutter R Advances in MRI around metal J Magn Reson Imaging 2017 46 4 972 991 10.1002/jmri.25708 28342291

[22] Khodarahmi I Nittka M Fritz J Leaps in technology: advanced MR imaging after total hip arthroplasty Semin Musculoskelet Radiol 2017 21 5 604 615 10.1055/s-0037-1606135 29025189

[23] Diaz-Ledezma C Higuera CA Parvizi J Success after treatment of periprosthetic joint infection: a Delphi-based international multidisciplinary consensus Clin Orthop Relat Res 2013 471 7 2374 2382 10.1007/s11999-013-2866-1 23440616 PMC3676607

[24] Albano D Pansa S Messina C et al. MRI of total hip arthroplasty: technical aspects and imaging findings Insights Imaging 2024 15 1 152 10.1186/s13244-024-01717-5 38900339 PMC11189891

[25] Fritz J Lurie B Miller TT Potter HG MR imaging of hip arthroplasty implants Radiographics 2014 34 4 E106 32 10.1148/rg.344140010 25019450

[26] Galley J Sutter R Stern C Filli L Rahm S Pfirrmann CWA Diagnosis of periprosthetic hip joint infection using MRI with metal artifact reduction at 1.5 T Radiology 2020 296 1 98 108 10.1148/radiol.2020191901 32396046

[27] George J Kwiecien G Klika AK et al. Are frozen sections and MSIS criteria reliable at the time of reimplantation of two-stage revision arthroplasty? Clin Orthop Relat Res 2016 474 7 1619 1626 10.1007/s11999-015-4673-3 26689583 PMC4887348

[28] Ghanem E Azzam K Seeley M Joshi A Parvizi J Staged revision for knee arthroplasty infection: what is the role of serologic tests before reimplantation? Clin Orthop Relat Res 2009 467 7 1699 1705 10.1007/s11999-009-0742-9 19241115 PMC2690749

[29] Aali Rezaie A Goswami K Shohat N Tokarski AT White AE Parvizi J Time to reimplantation: waiting longer confers no added benefit J Arthroplasty 2018 33 6 1850 1854 10.1016/j.arth.2018.01.073 29605153

[30] Borsinger TM Resnick CT Werth PM Schilling PL Moschetti WE Does time to reimplantation after explant for prosthetic joint infection influence the likelihood of successful outcomes at 2 years? J Arthroplasty 2022 37 6 1173 1179 10.1016/j.arth.2022.02.025 35176456

[31] Akcaalan S Ozaslan HI Caglar C Şimşek ME Citak M Akkaya M Role of biomarkers in periprosthetic joint infections Diagnostics (Basel) 2022 12 12 12 10.3390/diagnostics12122958 36552965 PMC9777153

[32] Deirmengian C Kardos K Kilmartin P Cameron A Schiller K Parvizi J Diagnosing periprosthetic joint infection: has the era of the biomarker arrived? Clin Orthop Relat Res 2014 472 11 3254 3262 10.1007/s11999-014-3543-8 24590839 PMC4182392

[33] Li B Chen F Liu Y Xu G Synovial Fluid α-Defensin as a biomarker for peri-prosthetic joint infection: a systematic review and meta-analysis Surg Infect (Larchmt) 2017 18 6 702 710 10.1089/sur.2017.006 28686144

[34] Zhang Z Cai Y Bai G et al. The value of calprotectin in synovial fluid for the diagnosis of chronic prosthetic joint infection Bone Joint Res 2020 9 8 450 456 10.1302/2046-3758.98.BJR-2019-0329.R2 32832073 PMC7418777

[35] Bai G Zeng H Li W The application of sonication in the diagnosis of peri⁃prosthetic infection after artificial joint replacement Chin J Orthop 2014 34 659 663 10.3760/CMA.J.ISSN.0253-2352.2014.06.010

[36] Hollyer I Ivanov D Kappagoda S Lowenberg DW Goodman SB Amanatullah DF Selecting a high-dose antibiotic-laden cement knee spacer J Orthop Res 2023 41 7 1383 1396 10.1002/jor.25570 37127938

[37] van Vugt TAG Arts JJ Geurts JAP Antibiotic-loaded polymethylmethacrylate beads and spacers in treatment of orthopedic infections and the role of biofilm formation Front Microbiol 2019 10 1626 10.3389/fmicb.2019.01626 31402901 PMC6671866

[38] Huang C Chen Y Ding H et al. Metal artifact reduction sequences MRI: a useful reference for preoperative diagnosis and debridement planning of periprosthetic joint infection J Clin Med 2022 11 15 4371 10.3390/jcm11154371 35955986 PMC9369276

[39] Albano D Messina C Zagra L et al. Failed total hip arthroplasty: diagnostic performance of conventional MRI features and locoregional lymphadenopathy to identify infected implants J Magn Reson Imaging 2021 53 1 201 210 10.1002/jmri.27314 32830902

[40] Jiang M He C Feng J et al. Magnetic resonance imaging parameter optimizations for diagnosis of periprosthetic infection and tumor recurrence in artificial joint replacement patients Sci Rep 2016 6 1 36995 10.1038/srep36995 27841342 PMC5107991

[41] Plesniar J Breit HC Clauss M Donners R Diagnosing periprosthetic hip joint infection with new-generation 0.55T MRI Eur J Radiol 2024 176 111524 10.1016/j.ejrad.2024.111524 38851014

[42] He C Lu Y Jiang M Feng J Wang Y Liu Z Clinical value of optimized magnetic resonance imaging for evaluation of patients with painful hip arthroplasty Chin Med J (Engl) 2014 127 22 3876 3880 10.3760/cma.j.issn.0366-6999.20141911 25421184

